# Evidence for susceptibility genes to familial Wilms tumour in addition to *WT1, FWT1* and *FWT2*

**DOI:** 10.1054/bjoc.2000.1283

**Published:** 2000-06-15

**Authors:** E A Rapley, R Barfoot, C Bonaïti-Pellié, A Chompret, W Foulkes, N Perusinghe, A Reeve, B Royer-Pokora, V Schumacher, A Shelling, J Skeen, S de Tourreil, A Weirich, K Pritchard-Jones, M R Stratton, N Rahman

**Affiliations:** Sections of Cancer Genetics, Paediatrics, Institute of Cancer Research, 15 Cotswold Ruad, Sutton, Surrey, SM2 5NG, UK; INSERM U521, Institut Gustav-Roussy, 39 rue Camille Desmoulins, Villejuif Cedex, 94805, France; Department of Medicine, Division of Medical Genetics and Division of Human Genetics, McGill University, 1650 Cedar Avenue, Montreal, Canada; Department of Biochemistry, University of Otago, Dunedin, New Zealand; Institut für Humangenetik und Anthropologie, Postfach 10 10 07, Düsseldorf, D-40001, Germany; Department of Obstetrics and Gynaecology, National Women’s Hospital, Auckland, New Zealand; Department of Paediatric Oncology, Starship Children’s Hospital, Auckland, New Zealand; Department of Haematology and Oncology, Children’s Hospital, University of Heidelberg, Im Neuenheimer Feld 150, Heidelberg, 69120, Germany

**Keywords:** Wilms tumour, *FWT1*, *FWT2*, familial predisposition

## Abstract

Three loci have been implicated in familial Wilms tumour: *WT1* located on chromosome 11p13, *FWT1* on 17q12-q21, and *FWT2* on 19q13. Two out of 19 Wilms tumour families evaluated showed strong evidence against linkage at all three loci. Both of these families contained at least three cases of Wilms tumour indicating that they were highly likely to be due to genetic susceptibility and therefore that one or more additional familial Wilms tumour susceptibility genes remain to be found. © 2000 Cancer Research Campaign


					Wilms tumour (WT) is an embryonal tumour of the kidney that
affects 1 in 10 000 children and accounts for 8% of all childhood
cancers (Stiller and Parkin, 1990). In 1-2% of cases the disease
clusters in families in which susceptibility to WT appears to be
predominantly inherited as an autosomal dominant trait with
incomplete penetrance (Breslow et al, 1996).
The genetics of familial WT is complex and at least three loci
predisposing to familial WT have been proposed. WT1 is a tumour
suppressor gene on chromosome 11p13. Constitutional WT1 muta-
tions have been documented in four families with more than one
case of WT (Yunis and Ramsay, 1980; Pelletier et al, 1991;
Kaplinsky et al, 1996; Pritchard-Jones et al, 2000). In all but one
(Kaplinsky et al, 1996), the WT1 mutation was associated with
congenital malformations, either urogenital abnormalities in males
and/or aniridia. WT1 has been excluded as the susceptibility gene
in several WT families in which no congenital abnormalities were
observed (Grundy et al, 1998; Huff et al, 1988; Schwartz et al,
1991; Baird et al, 1994).
We have mapped a familial WT susceptibility gene on chromo-
some 17q12-q21, designated FWT1, by genetic linkage analysis of
a large family of French-Canadian descent (MON 480) (Rahman
et al, 1996). The existence of this locus has been confirmed by
analysis of additional affected members from MON 480 and a
second unrelated pedigree (K1104) with seven cases of WT
(Rahman et al, 1998). WT cases in FWT1-linked pedigrees tend to
be diagnosed at a later age than non-familial cases (Rahman et al,
1998) and analyses of WT from MON 480 have demonstrated that
loss of the wild-type FWT1 allele, inherited from the non-mutation
carrying parent, does not occur (Rahman et al, 1997). Therefore,
FWT1 is unlikely to be a classical tumour suppressor gene.
Recently, an additional familial WT susceptibility gene (FWT2)
located on chromosome 19q13, has been proposed (McDonald et
al, 1998). The evidence in favour of this locus is not conclusive.
Furthermore, in families that were unlinked to the putative FWT2,
data at WT1 and FWT1 were not provided (McDonald et al, 1998).
It is currently unclear whether WT1, FWT1 and FWT2 account for
all familial WT predisposition, or whether additional familial WT
susceptibility genes are likely to exist. In this study we have
evaluated a set of WT families for the contribution of FWT2 and
have assessed the likelihood of the existence of additional WT
susceptibility genes.
MATERIALS AND METHODS
WT families
Families with two or more verified cases of WT were identified
from the UK, Canada, France, Germany, Switzerland, New
Zealand and USA. Permission for the study was given by the
Review Boards/Ethics Committee and informed consent was
obtained from the patient or parent as appropriate.
Microsatellite analysis
Genomic DNA was prepared from whole blood, from immortal-
ized lymphoblastoid cell lines and from fixed paraffin-embedded
tumour sections using standard techniques. Genotyping using
polymorphic microsatellite repeats was performed by standard
polymerase chain reaction (PCR) amplification with one primer
end-labelled using T4 polynucleotide kinase and [32
P]ATP.
PCR products were electrophoresed through 6% denaturing
polyacrylamide gels and the gel was exposed to autoradiography
film for 1-16 h.
Evidence for susceptibility genes to familial Wilms
tumour in addition to WT1, FWT1 and FWT2
EA Rapley1
, R Barfoot1
, C Bonaiti-Pellie2
, A Chompret2
, W Foulkes3
, N Perusinghe1
, A Reeve4
, B Royer-Pokora5
,
V Schumacher5
, A Shelling6
, J Skeen7
, S de Tourreil3
, A Weirich8
, K Pritchard-Jones9
, MR Stratton1
and N Rahman1
Sections of 1
Cancer Genetics and 9
Paediatrics, Institute of Cancer Research, 15 Cotswold Ruad, Sutton, Surrey SM2 5NG, UK; 2
INSERM U521, Institut
Gustav-Roussy, 39 rue Camille Desmoulins, 94805 Villejuif Cedex, France; 3
Department of Medicine, Division of Medical Genetics and Division of Human
Genetics, McGill University 1650 Cedar Avenue, Montreal, Canada; 4
Department of Biochemistry, University of Otago, Dunedin, New Zealand; 5
Institut fur
Humangenetik und Anthropologie, Postfach 10 10 07, D-40001 Dusseldorf, Germany; 6
Department of Obstetrics and Gynaecology, National Women's Hospital,
Auckland, New Zealand; 7
Department of Paediatric Oncology, Starship Children's Hospital, Auckland, New Zealand; 8
Department of Haematology and
Oncology, Children's Hospital, University of Heidelberg, Im Neuenheimer Feld 150, 69120 Heidelberg, Germany
Summary Three loci have been implicated in familial Wilms tumour: WT1 located on chromosome 11p13, FWT1 on 17q12-q21, and FWT2
on 19q13. Two out of 19 Wilms tumour families evaluated showed strong evidence against linkage at all three loci. Both of these families
contained at least three cases of Wilms tumour indicating that they were highly likely to be due to genetic susceptibility and therefore that one
or more additional familial Wilms tumour susceptibility genes remain to be found. (c) 2000 Cancer Research Campaign
Keywords: Wilms tumour; FWT1; FWT2; familial predisposition
177
Received 30 July 1999
Revised 2 March 2000
Accepted 7 March 2000
Correspondence to: N Rahman, Institute of Medical Genetics, University
Hospital of Wales, Cardiff CF4 4XW, UK. E-mail: nazneen@icr.ac.uk
British Journal of Cancer (2000) 83(2), 177-183
(c) 2000 Cancer Research Campaign
doi: 10.1054/ bjoc.2000.1283, available online at http://www.idealibrary.com on
Three markers were used to evaluate linkage to WT1. The
marker order determined from LDB (Collins et al, 1996) is
centromere-D11S904-2.5 cM-D11S4154-3.5 cM-D11S907-
telomere. WT1 is located between D11S4154 and D11S907. At
least six markers were used to evaluate linkage to FWT1.
Additional markers were analysed to generate informative data
when required. The marker order determined from LDB is
centromere-D17S946/D17S250- 2.5 cM-THRA1-1 cM-D17S8001
cM-D17S579-2.0 cM-D17S806-4 cM-D17S588- 2 cM-D17S1820-
telomere. At least six markers were examined to determine linkage
to FWT2. The marker ordered determined from Genethon marker
map (Dib et al, 1996) is centromere-D19S571-4 cM-D19S921-2.0
cM-D19S572-1.0 cM-D19S924-3.0 cM- D17S254/D19S418-3.0
cM-D19S926-1.0 cM-D19S891-telomere.
Statistical analysis
Genetic linkage analysis was performed using the FASTLINK
program (Cottingham et al, 1993). Familial WT was modelled as a
rare dominant (q = 0.000001) with a penetrance of 30% (Rahman
et al, 1996). Allele frequencies were calculated from 15 unrelated
individuals. Multipoint LOD scores were generated using two
informative markers from each chromosome haplotype. When
analysing family HPN12 the marriage loop was broken at ID1460,
using the makeped component of the LINKAGE package.
RESULTS
Of 13 previously published families with two or more cases of
WT, two families (WILMS 7 (Figure 1A) and FAMILY M
(Figure 1D) are highly unlikely to be due to either FWT1 or WT1
mutations (Rahman et al, 1998). Both families are unlinked at
FWT1. WILMS 7 is unlinked at WT1 (Rahman et al, 1998).
FAMILY M generates a small positive LOD score of 0.3 at WT1
(Table 1), but mutational screening by a combination of single
strand conformation polymorphism and direct sequencing did not
detect a predisposing WT1 mutation in this family (Baird et al,
1994). These two families were included in the current study. Six
previously unpublished families were also included. Three of
these (WILMS 12, WILMS 13 and HPN12) also show evidence
against linkage to FWT1 and WT1, and are illustrated in Figures
1B, C and E respectively. The remaining new families F2655
(uncle and nephew affected), F1124 (affected sib pair) and MON
948 (affected sib pair) were linked at either/both FWT1 and WT1
and are not shown. Therefore, five of our total series of 19 fami-
lies, WILMS 7, FAMILY M, WILMS 12, WILMS 13 and HPN12
are highly unlikely to be due to either WT1 or FWT1 mutations
(Figure 1, Table 1).
To evaluate the contribution of FWT2 and to assess the possi-
bility of additional familial WT susceptibility genes, the five
families that showed evidence against WT1 and FWT1 acting as
predisposition genes, were selected for analysis of markers in the
vicinity of FWT2. Multipoint LOD scores for these five families at
markers from the WT1, FWT1 and FWT2 regions are shown in
Table 1, and the segregating haplotypes of marker alleles in each
family are shown in Figure 1. Two of the five families (WILMS 7
and WILMS 12) show no evidence of a shared haplotype between
affected members at FWT2. The evidence against linkage is
reflected in the negative multipoint LOD scores (Table 1). In three
families (HPN12, WILMS 13 and FAMILY M) a chromosome 19q
marker haplotype is shared by the affected individuals. HPN12 has
a complex structure with the two affected individuals being related
through both parents. The multipoint analysis yields a maximum
LOD score of 1.00 at  = 0. WILMS 13 is an uncle/nephew pedi-
gree and generates a LOD score of 0.16. FAMILY M contains an
affected mother and two affected children and generates a LOD
score of 0.25. The WT from ID301 in WILMS 13 and ID302 in
FAMILY M showed loss of heterozygosity (LOH) at all markers
tested on chromosome 19q. In each tumour, the haplotype lost was
the one not linked to the disease in the family (Figure 1C, D).
DISCUSSION
Of 19 families with two or more individuals affected by WT, five
are unlikely to be due to mutation of either WT1 or FWT1. One of
these five families, HPN12 generated a multipoint LOD score of
1.00 at chromosome 19q13. Whilst not providing unambiguous
confirmation of its existence, this result suggests that the previous
localization of FWT2 to chromosome 19q may be correct. Two
further small families (WILMS 13 and FAMILY M) are consistent
with linkage to FWT2. In both families one WT showed somatic
loss of the haplotype that is not linked to the disease in the family.
Although this would be consistent with the conventional model of
a tumour suppressor gene, previous analyses of WT from families
putatively linked to FWT2 revealed that none of seven tumours
showed wild-type allele loss (McDonald et al, 1998). The signifi-
cance of the allele loss in WILMS 13 and FAMILY M is therefore
unclear. Moreover, as both families consist of few, closely related
individuals, linkage to chromosome 19q13 may have occurred by
chance and the WT predisposition gene in these families may well
be located elsewhere in the genome.
178 EA Rapley et al
British Journal of Cancer (2000) 83(2), 177-183 (c) 2000 Cancer Research Campaign
Table 1 Multipoint LOD scores for three familial WT loci, WT1, FWT1 and FWT2
Family Multipoint LOD score at  = 0
WT1 FWT1 FWT2
D11S904-6 cM-D11S907 D17S250-12.5 cM-D17S1820 D19S921-9.0 cM -D19S926
WILMS 7 -4.84 -4.85 -4.77
WILMS 12 -5.42 -5.43 -5.43
WILMS 13 -0.21 -5.17 0.16
FAMILY M 0.30 -5.32 0.25
HPN12 -4.35a
-4.27b
1.00c
a
Multipoint analysis using D11S4154-3.5 cM-D11S907. b
Multipoint analysis using D17S946-0.0 cM-D17S250-10.5 cM-D17S588
c
Multipoint analysis using D19S921-6.0 cM-D19S254-4.0 cM-D19S891
Evidence for a further familial WT gene 179
British Journal of Cancer (2000) 83(2), 177-183
(c) 2000 Cancer Research Campaign
971
9
9
9
9
9 9
4
4
9
4
7
7
7
7
7
4
4
4
4 4
4
4
4
D11S904
D11S4154
D11S907
D11S904
D11S4154
D11S907
D11S904
D11S4154
D11S907
3
3
3 3 3
1 1
5
5
5
5
5
4
15
15
9 9 9 9
9
9
9
6
6
6
6 6
15
4
(9)
2
2
2
2
2
15 15
5
5
6
5
2
15
16
16 14
15
(9)
2
2
978
977 973 970
975
971
977
982 975
971
977
982
978
975 974
973 970
974
978 973 970
974
982
WT 3
WT 3 WT 0.5
WT 3 WT 0.5 WT 1.8
WT 0.5
8
WT 1.8
11
11
11
10
15
15
10
10
10
10
10
7
4
4
4
4
4
6
5 5
10
10
10
7
7
7
7
7
1
1 1
1
1
4
6
6
6
10
10
10 10
10
10 10
14
20
17
17
12
12
12
7
2
2
2 2
11
10
17
12
13
12
3
3
3 3
3
3
3 3
3
3
9
9
6
10
14
12
D17S250
THRA1
D17S579
D17S806
D17s588
D17S1820
D17S250
THRA1
D17S579
D17S806
D17s588
D17S1820
D17S250
THRA1
D17S579
D17S806
D17s588
D17S1820
8
4
3
9
9
20
8
8
8
8
8
7
7
9
9
9
1
10
2
3
8
9
5
7
1
1
2
7
9
9
9
9
9
5
7
6
3
3
9
11
11
6
10
3
3
9
11
11
1
2
8
8
9
11
1
2
8
8
9
11
11
4
4
1
9
11
15
13
1
1
3
3
13
15
15
8
WT 1.8
D19S921
D19S571
D19S572
D19S924
D19S926
D19S418
D19S921
D19S571
D19S572
D19S924
D19S926
D19S418
D19S921
D19S571
D19S572
D19S924
D19S926
D19S418
A
180 EA Rapley et al
British Journal of Cancer (2000) 83(2), 177-183 (c) 2000 Cancer Research Campaign
D11S904
D11S4154
D11S907
D11S904
D11S4154
D11S907
D11S904
D11S4154
D11S907 1
1
1
1
1
1
2
4
4 4
4
7
5
5
5
5
5
6
1
2
2
4
4
4
4
7
9
9 9
7
7 7
7
7
6
6
6
6
6
10
6 5
6
5
5 5 5
5
3
8
8
3
8
8
8
8
8
8
8
2
10
11
11 11
17
13
16
15
16
4
4
4
5
5
5 5
5
8
5
4
5
6
6
6
6
5
7
8
11
16
12
10
12
2
2
1
1
1
1
1
1
7
7
11
10
2
4
8
5
3
3
9
9
11
10
10
10
11
11 11
9
9
13
13
15 15
12
6
8
10
10
10
10
10
17
2
3
3
3
5
5
6
10
15
3
3
2
7
7
2
2
2
4
4
4
4
7
5
6
3
3
3
3
4
1 1 1
1
1
5
10
11
7
1
9 9 9
13 13
3
3
3
3
9
7
6
8
13
16 16
3
9
7
6
8
8
8
13
16
3
5
3
8
5
3
8
8
8
5
8
8
WT 0.75
WT 8
WT 2.3
D17S250
THRA1
D17s800
D17S579
D17s 806
D17S588
D17S1820
D17S250
THRA1
D17s800
D17S579
D17s806
D17S588
D17S1820
D17S250
THRA1
D17s800
D17S579
D17s806
D17S588
D17S1820
WT 0.75
WT 8
WT 2.3
D19S571
D19S921
D19S572
D19S924
D19S418
D19S926
D19S571
D19S921
D19S572
D19S924
D19S418
D19S926
D19S571
D19S921
D19S572
D19S924
D19S418
D19S926
WT 0.75
WT 8
WT 2.3
B
Evidence for a further familial WT gene 181
British Journal of Cancer (2000) 83(2), 177-183
(c) 2000 Cancer Research Campaign
201
301
301
301
202 203 201
201
202
202
203
D11S904
D11S4154
D11S907
D11S904
D11S4154
D11S907
3
3
7
3 3
10
11
14
10
12 12
3
3
3
10
10
13
13
4
4
4
6
6
4
3
3
3
10
11
11
11
11
14
15
5
5
6
6
6
1
4
10
10
16
10
15
5
6
1
1
4
10
10
10
15
12
10
4
4
4
6 8
3
3
3
3
13
11
11
1 1
8
4 4
7
3
1
9
9
9
9
9
9
9
3
3
11
4
9
9
9
15
10
12
7
7
7
8
7
3
6
7
17
5
5
17
8
6
3
3
7
17
8
6
WT 1.25
WT 1.25
301
WT 1.25
WT 1.5 WT 1.5
203
WT 1.5
D17S250
THRA1
D17S579
D17S806
D17s588
D17S1820
D17S250
THRA1
D17S579
D17S806
D17s588
D17S1820
D19S921
D19S572
D19S924
D19S926
D19S418
D19S921
D19S572
D19S924
D19S926
D19S418
D19S921
D19S572
D19S924
D19S926
D19S418
tumour
-
-
-
-
D11S904
D11S4154
D11S907
D11S904
D11S4154
D11S907
D17S250
THRA1
D17S800
D17S579
D17S806
D17S588
D17S1820
D17S250
THRA1
D17S800
D17S579
D17S806
D17S588
D17S1820
D19S571
D19S921
D19S572
D19S924
D19S418
D19S926
D19S571
D19S921
D19S572
D19S924
D19S418
D19S926
D19S571
D19S921
D19S572
D19S924
D19S418
D19S926
202
301 302 302
201
WT 0.5
202 201
WT 0.5
202 201
WT 0.5
WT 0.4
301
WT 0.7
WT 0.7 WT 0.4 302
301
302
301
WT 0.7
WT 0.4
7
9
3
7
7
7
7
7
7
7
7
9
3
7
9
9
6
9
9 9
9
9
9
3
3
3
3
17
6
5
5
7
3
3
4
3
3
4
15
15
8 8
8
8 8
8
8
8
4
4
15
11
11 11
11
11
14
10 10 10
10
8
4
4
4
1
1 1
9
8
1
9
5
8
11 11
10
10
10
1
2
2 2
6
6
9
3
8
11
1
6
9
3
8
11
1
6
6
9
9
14
10
4
4
4
7
3
9
8
1
4
7
3
9
8
1
7
3
9
8
1
4
2
9
9
5
5
5
15
15
7
7
7
11
5
15
8
10
2
tumour tumour
-
-
-
-
-
C
D
182 EA Rapley et al
British Journal of Cancer (2000) 83(2), 177-183 (c) 2000 Cancer Research Campaign
D11S904
D11S4154
D11S907
2054
3 3
(7)
2053
7 16
(7)
1461
7 3
6
1460
1 1
6
3 6
4
3 6
4
1898
7 3
7
6 5
7
WT 3.25
D11S904
D11S4154
D11S907
1459
1 7
6
3 3
6
WT 2
D17S946
D17S250
THRA1
D17S800
D17S579
D17S806
D17S588
D17S1820
D17S946
D17S250
THRA1
D17S800
D17S579
D17S806
D17S588
D17S1820
2054
1898
8
8
3
10
9
9
9
9 9 9
9
9
5
5
5
7
7
7
(7)
(12)
(7)
7
7
7 7
7
7
7
7
7
7
7
1
5
5
5
6
6
6
6
6 6 6 1
6
6
6
14
14
10
10
10
11
11 11
11
11
11
2053
2
2
3
3
3 3 3 3 2
3
3
3
3
15
15
15
8
8
8 8 8
4
4
4
4
4
4
4
4
14 14 14
10
12
12
7
7
7
7
1
11
12
12 12
12
7
7
6
3
4
12
12
7
7
6
3
4
12
12
12
12
10 11
10
10
10
5
5
5
5
5
7
7
1
6
11
2
5
5
5
8
4
4
14
10
10
10
11
10
9
9
9
3
8
14
10
10
1461
1459
1460
WT 2
WT 3.25
D19S921
D19S572
D19S924
D19S252
D19S418
D19S926
D19S891
D19S921
D19S572
D19S924
D19S252
D19S418
D19S926
D19S891
2054
1898
2053 1461
1459
1460
WT 3.25 WT 2
Figure 1 Pedigrees of five WT families in which the disease is unlikely to be due to mutations in either FWT1 or WT1. Closed symbol WT, open symbol with
dot obligate carrier. The number after WT is the age at diagnosis. Haplotypes are shown by patterned bars. (A) WILMS 7; (B)WILMS 12; (C)WILMS 13;
(D)FAMILY M; (E) HPN12
E
Of the five families highly unlikely to be due to WT1 or FWT1,
two also showed strong evidence against linkage to markers in the
vicinity of FWT2. As there are at least three cases of WT in each of
these families, they are highly likely to be due to an underlying
genetic predisposition and therefore strongly suggest the existence
of at least one further familial WT susceptibility gene. Although
only two of the 19 families showed evidence against linkage at all
three known loci, many of the small familial clusters (such as
affected sib pairs) in the series of 19 families could have been
linked by chance to one or other locus. Indeed, of five families
with at least three cases of WT in our series, only one (FAMILY
M) is consistent with linkage at FWT2. Two (WILMS 7 and
WILMS 12) were unlinked at WT1, FWT1 and FWT2 and the two
remaining families showed clear evidence of linkage to FWT1
(MON 480 and K1104). It is thus possible that a substantial
proportion of susceptibility to familial WT that is not attributable
to WT1 or FWT1 is also not attributable to FWT2.
ACKNOWLEDGEMENTS
We would like to thank the families for their cooperation and the
many clinicians who have helped in the ascertainment of families,
including Dr Jon Pritchard, Dr Marie-France Tournade and Dr
Zappel. The work was supported by the Cancer Research
Campaign, the Medical Research Council of the UK and the
Cancer Research Society of Canada (Inc).
REFERENCES
Baird PN, Pritchard J and Cowell JK (1994) Molecular genetic analysis of
chromosome 11p in familial Wilms tumours. Br J Cancer 69: 1072-1077
Breslow NE, Olson J, Mokness J, Beckwith JB and Grundy P (1996) Familial
Wilms' tumour a descriptive study. Med Pediatr Oncol 27: 398-403
Collins A, Frezal J, Teague J and Morton NE (1996) A metric map of humans: 23
500 loci in 850 bands. Proc Natl Acad Sci USA 93: 14771-14775
Cottingham RW, Idury RM and Schaffer AA (1993) Faster sequential genetic
linkage computations. Am J Hum Genet 53: 252-263
Dib C, Faure S, Fizames C, Samson D, Drouot N, Vignal A, Millasseau P, Marc S,
Hazan J, Seboun E, Lathrop M, Gyapay G, Morissette J and Weissenbach J
(1996) A comprehensive genetic map of the human genome based or 5264
microsatellites. Nature 380: 152-154
Grundy P, Koufos A, Morgan K, Li FP, Meadows AT and Cavenee WK (1988)
Familial predisposition to Wilms' tumour does not map to the short arm of
chromosome 11. Nature. 336: 374-376
Huff V, Compton DA, Chao LY, Strong LC, Geiser CF and Saunders GF (1988)
Lack of linkage of familial Wilms' tumour to chromosomal band 11p13.
Nature 336: 377-378
Kaplinsky C, Ghahremani M, Frishberg Y, Rechavi G, Pelletier J (1996) Familial
Wilms' tumor associated with a WT1 zinc finger mutation. Genomics 38:
451-453
McDonald JM, Douglass EC, Fisher R, Geiser CF, Krill CE, Strong LC, Virshup D
and Huff V (1998) Linkage of familial Wilms' tumor predisposition to
chromosome 19 and a two-locus model for the etiology of familial tumors.
Cancer Res 58: 1387-1390
Pelletier J, Bruening W, Li FP, Haber DA, Glaser T, Housman DE (1991) WT1
mutations contribute to abnormal genital system development and hereditary
Wilms' tumour. Nature 353: 431-434
Pritchard-Jones K, Rahman N, Gerrard M, Variend D and King-Underwood L (2000)
Familial Wilms tumour due to WT1 mutation: intronic polymorphism causing
artefactual constitutional homozygosity. J Med Genet (in press)
Rahman N, Arbour L, Tonin P, Renshaw J, Pelletier J, Baruchel S, Pritchard-Jones
K, Stratton MR, Narod SA (1996) Evidence for a familial Wilms' tumour gene
(FWT1) on chromosome 17q12-q21. Nat Genet 13: 461-463
Rahman N, Arbour L, Tonin P, Baruchel S, Pritchard-Jones K, Narod SA and
Stratton MR (1997) The familial Wilms' tumour susceptibility gene, FWT1,
may not be a tumour suppressor gene. Oncogene 14: 3099-30102
Rahman N, Abidi F, Ford D, Arbour L, Rapley E, Tonin P, Barton D, Batcup G,
Berry J, Cotter F, Davison V, Gerrard M, Gray E, Grundy R, Hanafy M, King
D, Lewis I, Ridolfi Luethy A, Madlensky L, Mann J, O'Meara A, Oakhill T,
Skolnick M, Strong L, and Stratton MR (1998) Confirmation of FWT1 as a
Wilms' tumour susceptibility gene and phenotypic characteristics of Wilms'
tumour attributable to FWT1. Hum Genet 103: 547-556
Schwartz CE, Haber DA, Stanton VP, Strong LC, Skolnick MH and Housman DE
(1991) Familial predisposition to Wilms tumor does not segregate with the
WT1 gene Genomics 10: 927-930
Stiller CA and Parkin (1990) International variations in the incidence of childhood
renal tumours. Br J Cancer 62: 1026-1030
Yunis JJ and Ramsay NK (1980) Familial occurrence of the aniridia-Wilms tumor
syndrome with deletion 11p13-14.1. J Pediatr 96: 1027-1030
Evidence for a further familial WT gene 183
British Journal of Cancer (2000) 83(2), 177-183
(c) 2000 Cancer Research Campaign

				
